# Analysis of Selected Risk Factors Depending on the Type of Cerebral Palsy

**DOI:** 10.3390/brainsci11111448

**Published:** 2021-10-30

**Authors:** Małgorzata Sadowska, Beata Sarecka-Hujar, Ilona Kopyta

**Affiliations:** 1 Department of Pediatric Neurology, Faculty of Medical Sciences in Katowice, Medical University of Silesia in Katowice, 40-752 Katowice, Poland; m.sadowscy@gmail.com (M.S.); ilonakopyta@autograf.pl (I.K.); 2 Department of Basic Biomedical Science, Faculty of Pharmaceutical Sciences in Sosnowiec, Medical University of Silesia in Katowice, 41-200 Sosnowiec, Poland

**Keywords:** cerebral palsy, children, risk factors

## Abstract

*Background:* Cerebral palsy (CP) is not a defined, separate disease classification, but a set of etiologically diverse symptoms that change with the child’s age. According to the up-to-date definition, CP is a group of permanent but not unchanging disorders of movement and/or posture and motor function, which are due to a nonprogressive interference, lesion, or abnormality of the developing/immature brain. CP is one of the most frequent causes of motor disability in children. The aim of the present study was to analyze whether selected risk factors may vary depending on particular types of CP. *Methods:* 181 children with CP (aged 4–17 years), hospitalized at the Department of Pediatrics and Developmental Age Neurology in Katowice in the years 2008–2016 were retrospectively analyzed in the present study. The assumed risk factors of CP were divided into two groups: 1—pre-conception and prenatal (mother’s age, family history of epilepsy, burdened obstetric history, mother’s systemic diseases, pregnancy order, multiple pregnancy, duration of pregnancy, bleedings from the genital tract during gestation, arterial hypertension during pregnancy, infections during pregnancy, preterm contractions, maintained pregnancy, premature rupture of membranes, abruptio placentae, and others), 2—perinatal and postnatal (mode of delivery, birth weight, Apgar score at the first and fifth minute, neonatal convulsions, respiratory failure, infections in neonatal and infant period, and intraventricular bleeding). The division into particular CP types was based on Ingram’s classification. *Results:* The following risk factors were the most frequent in the total group: respiratory failure, infections, intraventricular bleeding, and prematurity. Among the analyzed preconception and prenatal factors, the duration of pregnancy and preterm contractions during pregnancy significantly differentiated the subgroups of patients depending on the type of CP. The prevalence of almost all analyzed perinatal, neonatal, and infant-related risk factors (i.e., birth weight, Apgar score at the first and fifth minute, neonatal convulsions, respiratory failure, infections in neonatal and infant period, and intraventricular bleeding) significantly differed between CP types, apart from the mode of delivery. However, in multivariate regression, only intraventricular bleeding was an independent predictor for tetraplegic CP type when compared to joined extrapyramidal and ataxic types (OR = 2.801, *p* = 0.028). *Conclusions:* As CP is a syndrome of multifactorial etiology, the identification of CP risk factors entails the need for careful observation and comprehensive care of children in the risk group. The presence of certain risk factors may be a prognostic indicator for particular types of CP. The knowledge about the association between the risk factor(s) and the CP type could be a very useful tool for pediatricians looking after the child at risk of developmental disorders.

## 1. Introduction

Cerebral palsy (CP) is of particular importance among many diseases of the nervous system, being one of the most common physical disabilities of childhood [1,2,3,4,5,6]. CP is an “umbrella term”, i.e., it describes a group of permanent but not unchanging disorders of movement and/or posture and motor function, which are due to a nonprogressive interference, lesion, or abnormality of the developing/immature brain [7,8,9]. Its frequency is established at 1.5 to 3.0 per 1000 live-born children; however, these values may differ between selected groups of patients, depending on various risk factors [10]. CP is a set of etiologically diverse symptoms that change with the child’s age. A number of various factors may cause damage to the central nervous system at an early stage of its development. Potential risk factors for CP can be divided into:

- pre-conception (concerning the broadly defined health and living conditions of the mother) and prenatal (related to the course of pregnancy),
- perinatal, neonatal, and infant period-related risk factors [4,5,11,12,13,14,15,16,17,18,19,20,21,22,23,24,25,26,27,28,29,30].

The etiology of CP is multifactorial and various risk factors were previously established.

Van Lieshout et al. [31] concluded in the systematic review that reliable risk factors for developing CP include: chorioamnionitis, low arterial cord pH, essential hypertension during pregnancy, hypertension in the second half of pregnancy, bleeding from the genital tract after 20 weeks of pregnancy, preeclampsia, late preterm gestation age, intrauterine growth restriction, primiparity, low birth weight, low Apgar score, multiple gestation/births, fever during the delivery, wrapping of the umbilical cord around the neck, sepsis in the neonatal period, and hypoxic-ischemic encephalopathy [31].

Polish author Mieszczanek analyzed the most common causes of CP in a group of 445 babies with CP from western–southern Poland and compared them with the control group of infants born during the same period 1981–1998 in the same area [32]. In the author’s opinion, highly statistically significant CP risk factors were as follows: low birth weight, Apgar score below five points at the first minute after birth, risk of preterm labor, vaginal bleeding during pregnancy, prolonged (more than seven days) mechanical ventilation, and multiple pregnancy. The author also considered abnormalities in the course of previous pregnancies, the premature rupture of membranes, the mother’s age over 35 years, as well as the abnormal position of the fetus as significant risk factors for CP [32]. 

In turn, the Swedish cohort study demonstrated associations between potential antenatal and perinatal risk factors and cerebral palsy, depending on the gestational age: term (≥37 weeks), moderately or late preterm (32–36 weeks), and very preterm (<32 weeks) [20]. The authors conclude, that in term-born children, maternal obesity, small for gestational age, malformations, induction, and elective and emergency cesarean section, Apgar score < seven at five min and admission to neonatal care were significantly associated with CP. Among children born moderately or late preterm, small for gestational age, malformations, elective and emergency caesarean section, and admission to neonatal care were all associated with CP, whereas in children born very preterm, no factors were significantly associated with the outcome.

Recently, a genetic basis for the same cases of CP has also been suspected. A study by Zhu et al. [22] based on ten children with CP demonstrated that three pathways (i.e., axon guidance, transmission across chemical synapses, and protein–protein interactions at synapses) with twenty-three genes are highly correlated with the disease. In turn, a study by McMichael et al. [23] identified 61 de novo protein-altering variants in 44% of case (i.e., children with CP)–parent trios.

However, there are few studies analyzing the associations between potential risk factors and the clinical types of CP.

CP is a very heterogeneous set of motor disorders with many existing classifications, of which the most commonly applied include Ingram’s classification [33], Hagberg’s classification [34], and the classification proposed by the Surveillance of Cerebral Palsy in Europe (SCPE) [9]. 

The aim of the present study was to analyze the frequency of selected risk factors (pre-conception, prenatal, perinatal, neonatal, and infant period-related risk factors) in particular types of cerebral palsy according to Ingram’s classification in a group of children hospitalized at the Department of Paediatrics and Developmental Age Neurology in Katowice in the years 2008–2016.

## 2. Materials and Methods

### 2.1. Study Group

The present study is a clinical records-based case series with a retrospective analysis of exposure factors using a case-control approach. We evaluated 181 children (98 boys and 83 girls) with CP (aged 4–17 years). Medical records of patients hospitalized, i.e., admitted at the Department of Paediatrics and Developmental Age Neurology in Katowice between 2008 and 2016, were analyzed according to ICD-10 classification. We focused on G80 patients’ records from the hospital data system.

Table 1 shows the inclusion/exclusion criteria that were used to extract the most relevant records.

Division into CP types, i.e., diplegia, hemiplegia, tetraplegia, the extrapyramidal, ataxic, and mixed forms was based on Ingram’s classification [33]. We used the classical classification, which is rather “old”, but still popular and used both by practicing doctors as well as by scientists. We performed a retrospective study and during the period our patients were enrolled, the SCPE classification was just new and entering to be used. In the records of the patients included, Ingram’s classification was used and the usage of the SCPE classification to these patients was not always possible due to lacking some of the data.

The Local Ethical Committee gave their opinion on the study (decision No. KNW/0022/KB/179/16).

### 2.2. Potential Risk Factors for CP Type

Based on a thorough analysis of medical records, potential risk factors for CP have been identified and divided into two groups:

- preconception and prenatal risk factors (mother’s age, mother’s systemic diseases, burdened obstetric history, family history of epilepsy, the order of pregnancy, multiple pregnancy prevalence, bleedings from the genital tract during gestation, arterial hypertension in pregnancy, infections during pregnancy, premature contractions, maintained pregnancy, premature placental abruption, premature rupture of the membranes, and others)
- perinatal, neonatal, and infant-related risk factors (mode of delivery, birth weight, Apgar score at the first and fifth minute, neonatal convulsions, respiratory failure, intracranial bleedings, and infections in neonatal/infant period).

The term “maintained pregnancy” was used for cases when the pregnant woman was undergoing hormone therapy with progesterone.

The term “infections“ was used to describe the presence of any infection during pregnancy, the neonatal period, or in infancy, regardless of etiology (bacterial, viral, fungal) and symptomatology (urinary tract infection, respiratory tract infection, neuroinfection, or generalized infection).

In the present study, the frequency of potential risk factors for cerebral palsy was compared between particular CP types.

### 2.3. Statistical Analysis

Data were analyzed using STATISTICA 13.0 software (STATSOFT; Statistica, Tulsa, OK, USA). For categorical variables, absolute numbers (n) and relative numbers (%) were estimated. For numerical variables, mean value (M) and standard deviation (SD) were estimated. We used a stochastic independence χ^2^ test with Yates’s correction to compare categorical variables between CP types as well as an analysis of variance F test to compare numerical variables between CP types.

The multivariable polynomial logistic regression analysis was also performed. Dependent variable was type of CP. Predictors were preconception, prenatal, perinatal, neonatal, and infant-related risk factors which were significantly associated with CP types in univariate analyses. Selection of covariates was conducted using backward step-by-step method.

A value of *p* ≤ 0.05 was considered to be statistically significant.

## 3. Results

### 3.1. Characteristic of the Study Group

The present study is based on a patients’ sample, and the characteristics of the total group of included patients were presented in our previous research [35].

### 3.2. The Frequency of Preconception and Prenatal Risk Factors Depending on the Type of CP

Table 2 presents the prevalence of preconception and prenatal risk factors in different CP types.

The mean duration of pregnancy differed significantly between CP types (*p* < 0.001). The lowest length of pregnancy was observed for diplegia. Among the analyzed pre-conception and prenatal risk factors, only the duration of pregnancy and the occurrence of premature contractions differentiated particular forms of CP from one another. Diplegia was predominant in the case of children born between 28 and incomplete 32 weeks, i.e., very preterm infants, whereas hemiplegia, the ataxic, and mixed forms were most often observed in full-term children, i.e., born between 37 and incomplete 42 weeks. Premature contractions were prevalent in diplegia and tetraplegia.

### 3.3. The Frequency of Perinatal, Neonatal and Infant-Related Risk Factors Depending on CP Type

The prevalence of perinatal, neonatal, and infant-related risk factors in various types of CP is shown in Table 3.

Mean birth weight differed significantly between CP types (*p* < 0.001, *p* = 0.012, and *p* = 0.003, respectively). The lowest mean birth weight was observed in diplegic children while the highest was observed in hemiplegic children.

Among the peri- and postnatal risk factors, only the mode of delivery did not differentiate between particular forms of CP. On the other hand, birth weight, Apgar score at 1st and 5th minute after birth, the prevalence of neonatal seizures, respiratory failure, intraventricular bleeding, and infections in the neonatal and infant period were significantly different for particular types of CP.

In diplegia, very low and low birth weight prevailed, and in hemiplegia: birth weight 2500–4000 g. The severe condition (an Apgar score of 0–3) at 1st minute prevailed in children with the extrapyramidal form and tetraplegia; the medium condition (an Apgar score of 4–7) in diplegia, and the good condition (an Apgar score of 8–10) in hemiplegia.

At 5th minute after birth, the severe condition was most observed in the case of patients with the mixed form; the medium condition in patients with diplegia, tetraplegia, and the extrapyramidal form, and the good condition in patients with hemiplegia and the ataxic form.

**Figure 1:**
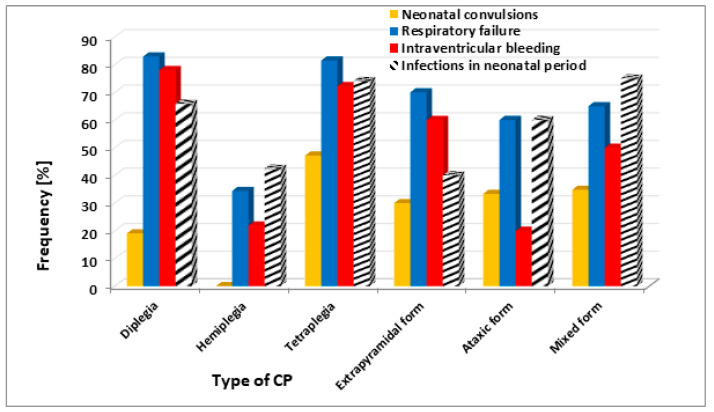
Frequency of neonatal convulsions, respiratory failure, intraventricular bleeding, and infections in neonatal period depending on the type of cerebral palsy.

Figure 1 demonstrates the frequency of neonatal convulsions, respiratory failure, intraventricular bleeding, and infections in the neonatal period in various types of CP.

Neonatal convulsions and infections occurred most frequently in tetraplegia and in the mixed type, while respiratory failure and intraventricular bleeding occurred most frequently in diplegia and tetraplegia.

In the multivariate polynomial logistic regression model, we modeled a probability of prevalence of one of the CP types versus the reference CP type. A reference CP type was joined extrapyramidal and ataxic CP. From the predictors significant in univariate analyses, Apgar score at 5th minute was not taken into account since data were missing in almost 28% of patients, as well as being positively correlated with Apgar score at 1st minute (r = 0.918, *p* < 0.001). From predictors significant in the univariate analysis, i.e., birth weight, duration of pregnancy, Apgar score at 1st minute, premature contractions, respiratory failure, infections, intraventricular bleeding, and neonatal convulsions, the independent predictor for tetraplegic CP type was only intraventricular bleeding (OR = 2.801, *p* = 0.028). The risk of tetraplegia was almost 3-fold higher than the risk of reference CP type when intraventricular bleeding occurred.

## 4. Discussion

Many publications are devoted to the assessment of risk factors for cerebral palsy, but most cohort studies analyze the CP risk in general terms, depending on the selected factor/factors [2,3,4,5,12,31,32,36,37,38,39].

In the present study, no significant statistical differences were found between the various types of CP in terms of: mother’s age, mother’s systemic diseases, burdened obstetric history, family history of epilepsy, the order of pregnancy, multiple pregnancy, as well as the course of pregnancy (except for premature contractions), whereas the duration of pregnancy and the presence of premature contractions differed between the CP types from one another.

However, significant differences were found among the perinatal, neonatal, and infant-related risk factors with regard to: birth weight, Apgar scores at the first and the fifth minute, the prevalence of neonatal convulsions, respiratory failure, intraventricular bleedings, and infections in the neonatal period. The mode of delivery did not differentiate between particular forms of CP.

There is little research comparing the frequency of risk factors in particular CP types. These studies differ in terms of the risk factors considered, which makes an objective comparison between the authors’ own results and those obtained by other researchers difficult or impossible to carry out. Ahlin et al. [13] attempted to analyze a number of very diverse factors, apart from prematurity, that could be important in the etiopathogenesis of CP. The authors found that both pre-, peri-, and postnatal factors may play a role in the pathogenesis of diplegia and tetraplegia; the risk of hemiplegia is increased mainly by prenatal factors. However, in their opinion, only perinatal factors contribute to the etiology of the dyskinetic form [13].

A Polish retrospective study by Kułak et al. [11], compared the CP risk factors before delivery, during childbirth, and in the neonatal period in 345 children with CP and in a control group consisting of 360 children without CP. Taking into account the distribution of CP type, the authors demonstrated no correlation between the CP type and the duration of pregnancy. Hemiplegia, diplegia, as well as tetraplegia were more common in full-term than in premature children.

In our study, diplegia was the most common in very preterm children, i.e., between 28 and incomplete 32 weeks (43%) and tetraplegia was the most common in moderately and late-preterm children, i.e., between 32 and incomplete 37 weeks, and in full-term children (36% each), whereas hemiplegia occurred in the vast majority of full-term children (66%).

The relationship between fetus age and the type of CP was assessed in a meta-analysis conducted by Himpens et al. [36]. Two main types of CP were analyzed: spastic type (unilateral spastic and bilateral spastic) and non-spastic type (dyskinetic and ataxic type). The spastic types prevailed both in the group of premature and full-term children. The non-spastic types were more common among full-term children (18%) as compared to preterm children (5%). Among the spastic forms, the bilateral spastic type occurred in more than two-thirds of the preterm children and in approximately half of the full-term children. On the other hand, a statistically significantly higher percentage of unilateral spastic CP was found among full-term children [36].

In our study, a different CP classification was used. However, the present results may be compared with those by Himpens et al. [36]. The bilateral spastic type corresponded to diplegia and tetraplegia in our study, which accounted for 72% of all preterm children. Hemiplegia corresponded to unilateral spasticity, which occurred in 13% of preterm children and in 31% of full-term children, and is partially consistent with the results from a meta-analysis conducted by Belgian researchers [36]. Kułak et al. [40] compared the diplegic and tetraplegic forms of CP. The authors analyzed the following risk factors: the sequence of pregnancies, duration of pregnancy, premature placental abruption, premature rupture of membranes, pre-eclampsia, delivery by cesarean section, very low and low birth weight, the occurrence of respiratory distress syndrome, and sepsis in newborns.

In our study, no difference was found between diplegia and tetraplegia in terms of the order of pregnancy, premature placental abruption, premature rupture of membranes, or the mode of delivery. Contrary to the studies by Kułak et al. [40], the birth weight was different in particular types of CP.

Mieszczanek [32] found that mechanically ventilated children most often developed diplegic (55%) or tetraplegic (38%) CP.

In the present study, we analyzed the prevalence of respiratory failure in children with various types of CP, and its prevalence was the highest in diplegia and tetraplegia, similarly to the study by Mieszczanek [32].

Significant data on the infant’s health is provided by the Apgar score, which reflects the viability of the baby in the first minutes after birth and is based on the assessment of heart rate, respiration, skin color, muscle tone, and response to stimuli [41]. The relationship between a low Apgar score and the risk of neonatal death or chronic neurological complications, including CP, has been the subject of numerous studies [42,43,44,45,46].

Norwegian researchers conducted an analysis of the relationship between cerebral palsy and the Apgar score at the fifth minute after birth in all children with normal and low birth weight born in Norway in 1986–1995 who survived the first year of life. They also examined the correlation between the infant’s Apgar score and particular spastic forms of CP: tetraplegia, diplegia, and hemiplegia. A low Apgar score (<7 points) correlated with all three spastic forms of CP, most clearly with tetraplegia [44].

Mieszczanek found no statistical correlation between the low Apgar score at the first minute after birth and the clinical form of CP [32].

In our study, an analysis of the Apgar score was performed both at the first and fifth minute after birth. The Apgar score in both cases differentiated the individual types of CP forms.

In the examined group, the Apgar score at the first minute after birth ranging from 0–3, defined as a severe neonatal condition, was most often observed in infants with the extrapyramidal form and tetraplegia, while the lowest score (from 0–3) was most often observed at the fifth minute in children with a mixed form. Scores from 4–7 at the first and fifth minute were most often noted in the case of children with diplegia (52% and 53%, respectively). On the other hand, scores from 8–10, assessed as the infant’s good condition, at both the first and fifth minute, characterized, in contrast to the Norwegian authors’ research, children with hemiplegia (75% and 72%, respectively). Children with the ataxic form were also characterized by a high Apgar score (from 8–10 points) both at the first and fifth minute after birth, but this group was very small (six children, i.e., 3.3% of the total group).

The results based on the analyzed group of patients have shown that the presence of neonatal convulsions, respiratory failure, intraventricular bleeding, and infections in the neonatal and infant period differentiated particular forms of CP. Neonatal convulsions were most often observed in patients with tetraplegia, respiratory failure, and intraventricular bleeding, in children with diplegia and tetraplegia, and infections in the neonatal and infant period, in children with tetraplegia and the mixed form of CP. We observed in multivariate regression that only intraventricular bleeding was an independent predictor for tetraplegic CP type (OR = 2.801, *p* = 0.028).

Kułak et al. [40] observed no statistically significant differences between diplegia and tetraplegia in terms of neonatal seizures or respiratory distress syndrome. The authors did not specifically analyze the respiratory failure syndrome, only the overall respiratory failure in relation to all forms of CP. A comparison of diplegia and tetraplegia indicates that respiratory failure was very similar in both CP types (83% and 81%, respectively).

An early life inflammation, which causes white matter damage during a developmentally vulnerable period, is one of the predictors of various long-term neurodevelopmental outcomes. Various antenatal and postnatal factors can trigger perinatal inflammation. Multiple episodes of inflammation are more injurious to the brain than a single episode. Among the numerous factors associated with perinatal inflammation are intrauterine infection, fetal growth restriction, severe brain hemorrhage, prolonged mechanical ventilation, necrotizing enterocolitis, and sepsis [19,24]. Bear and Wu found that not only intrauterine but also extra-uterine maternal infections during pregnancy were associated with a higher risk of CP [30]. Kuban et al. evaluated the associations between neonatal systemic inflammation in extremely preterm children and cerebral palsy at 24 months [28]. The study proved that neonatal systemic inflammation was associated with an increased risk of each form of cerebral palsy (tetraparesis, diparesis, and hemiparesis).

In the ELGAN (Extremely Low Gestational Age Newborn) Study, American investigators evaluated the contribution of both antenatal and postnatal inflammation to the risk of neurodevelopmental disorders. They defined antenatal inflammation as histologic inflammation in the placenta. To evaluate postnatal inflammation, they measured the concentrations of seven inflammation-related proteins in blood obtained on postnatal days 1, 7, and 14 from 763 children born before 28 weeks of gestation (the authors defined postnatal inflammation as a protein concentration in the highest quartile over at least two days). Children with both antenatal inflammation and postnatal systemic inflammation were more likely to develop brain ultrasound indicators of cerebral white matter damage and developmental impairments at 24 months of age than were children with only placenta inflammation or only neonatal systemic inflammation. The authors concluded that placental inflammation and postnatal systemic inflammation were together associated with a higher risk of all structural and clinical indicators of white matter damage, except spastic hemiparesis [27].

In our study, we did not analyze the inflammation mediators but the overall presence of various infections in the neonatal and infant periods, which were most often observed in children with tetraplegia and the mixed form of CP.

The present study has some limitations. First, the study is retrospective, which is the reason for the limited data and factors we could evaluate; therefore, we focused on those available in patients’ records. Second, the study analyzed a relatively low number of patients. Third, the children with extrapyramidal and cerebellar types were underrepresented.

Despite the limitations of the present study, from our point of view, it would be valuable to continue the research on risk factors for particular types of CP as a prospective one with inclusion/exclusion criteria established clearly at the beginning of the study. It would also be of great interest to make a unified plan for the diagnostic process; for example, time and type of neuroimaging.

## 5. Conclusions

Cerebral palsy is a syndrome with a multifactorial etiology. The most common risk factors in the total study group were as follows: respiratory failure, intraventricular bleeding, infections in the neonatal period, and prematurity. However, intraventricular bleeding was the only independent predictor for tetraplegia when compared to joined extrapyramidal and ataxic types.

The presence of certain risk factors may be a prognostic indicator for the particular type of cerebral palsy.

The knowledge about the correlation between the risk factor(s) and the CP type could be a very useful tool for pediatricians looking after children at risk of developmental disorders.

## Figures and Tables

**Table 1:** Inclusion/exclusion criteria used to extract the most relevant records from the hospital data system.

Inclusion Criteria	Exclusion Criteria
Age between 4 and 17 years	Clinical features of progressive encephalopathies
The diagnosis of cerebral palsy verified by an experienced pediatric neurologist	
Available neuroimaging: magnetic resonance imaging (MRI) or computed tomography (CT) results	Metabolic inborn errors

**Table 2:** Frequency of preconception and prenatal risk factors depending on CP type.

	Total Group	Hemiplegia	Diplegia	Tetraplegia	Mixed Form	Extrapyramidal Form	Ataxic Form	*p*
	*n* = 181	*n* = 39	*n* = 44	*n* = 57	*n* = 25	*n* = 10	*n* = 6	
Mother’s age, (years), M ± SD	27.9 ± 6.3	28.2 ± 5.7	27.1 ± 6.4	28.4 ± 6.6	26.7 ± 7.2	30.9 ± 5.4	27.2 ± 2.9	0.525
Mother’s age, *n* (%) (missing data *n* = 14)								0.648
<20 years old	13 (7.8)	0 (0.0)	5 (12.2)	5 (9.3)	3 (13.0)	0 (0.0)	0 (0.0)	
20–34 years old	123 (73.6)	28 (82.3)	30 (73.2)	38 (70.4)	15 (65.2)	7 (70.0)	5 (100.0)	
≥35 years old	31 (18.6)	6 (17.6)	6 (14.6)	11 (20.4)	5 (21.7)	3 (30.0)	0 (0.0)	
Mother’s systemic diseases *, *n* (%) (missing data *n* = 9)	24 (13.9)	7 (18.4)	7 (17.5)	6 (10.9)	1 (4.2)	2 (20.0)	1 (20.0)	0.575
Burdened obstetric history, *n* (%) (missing data *n* = 6)	42 (24.0)	10 (27.0)	7 (17.1)	15 (26.3)	7 (29.2)	2 (20.0)	1 (16. 7)	0.821
Family history of epilepsy, *n* (%) (missing data *n* = 5)	10 (5.7)	0 (0.0)	1 (2.4)	4 (7.0)	3 (12.0)	2 (20.0)	0 (0.0)	0.100
The order of pregnancy, *n* (%)								0.292
(missing data *n* = 5)								
I	78 (44.3)	14 (36.8)	15 (35.7)	28 (50.0)	14 (56.0)	4 (40.0)	3 (60.0)	
II	45 (25.6)	9 (23.7)	11 (26.2)	13 (23.2)	7 (28.0)	3 (30.0)	2 (40.0)	
III	30 (17.0)	9 (23.7)	11 (26.2)	9 (16.1)	0 (0.0)	1 (10.0)	0 (0.0)	
IV	12 (6.8)	3 (7.9)	2 (4.8)	3 (5.4)	3 (12.0)	1 (10.0)	0 (0.0)	
V–VIII	11 (6.2)	3 (7.9)	3 (7.1)	3 (5.4)	1 (4.0)	1 (10.0)	0 (0.0)	
Multiple pregnancy, *n* (%)								0.908
(missing data *n* = 2)	14 (7.8)	3 (7. 9)	4 (9.1)	5 (8.9)	2 (8.0)	0 (0.0)	0 (0.0)	
Duration of pregnancy (full weeks), M ± SD	35.1 ± 4.8	37.5 ± 3.4	31.9 ± 4.4	35.2 ± 4.5	36.4 ± 5.1	34.5 ± 5.5	36.8 ± 5.8	**<0.001**
Duration of pregnancy, *n* (%)								**<0.001**
(missing data *n* = 3)								
<28 weeks	14 (7.9)	0 (0.0)	6 (13.6)	3 (5.4)	2 (8.3)	2 (20.0)	1 (16.7)	
28–<32 weeks	35 (19.7)	3 (7.9)	19 (43.2)	11 (19.6)	2 (8.3)	0 (0.0)	0 (0.0)	
32–<37 weeks	46 (25.8)	9 (23.7)	9 (20.4)	20 (35.7)	4 (16. 7)	4 (40.0)	0 (0.0)	
37–<42 weeks	80 (44.9)	25 (65.8)	10 (22.7)	20 (35.7)	16 (66. 7)	4 (40.0)	5 (83.3)	
≥42 weeks	3 (1.7)	1 (2.6)	0 (0.0)	2 (3.6)	0 (0.0)	0 (0.0)	0 (0.0)	
The course of pregnancy, *n* (%) (missing data *n* = 11)								
Hypertension	15 (8.8)	3 (9.8)	4 (9.8)	7 (12.3)	1 (4.3)	0 (0.0)	0 (0.0)	0.716
Genital tract bleeding	24 (14.1)	3 (8.8)	9 (21.9)	8 (14.0)	2 (8.7)	2 (20.0)	0 (0.0)	0.48
Infections	33 (19.4)	7 (20.6)	8 (19.5)	10 (17.5)	5 (21.7)	1 (10.0)	2 (40.0)	0.829
Maintained pregnancy	37 (21.8)	6 (17.6)	12 (21.0)	12 (21.0)	4 (17.4)	2 (20.0)	1 (20.0)	0.851
Premature contractions	30 (17.6)	1 (2.9)	12 (29.3)	14 (24.6)	1 (4.3)	1 (10.0)	1 (20.0)	**0.016**
Premature placental abruption	18 (10.6)	2 (5.9)	6 (14.6)	9 (15.8)	0 (0.0)	1 (10.0)	0 (0.0)	0.268
Premature rupture of the membranes	28 (16.5)	3 (8.8)	11 (26.8)	6 (10.5)	5 (21.7)	3 (30.0)	0 (0.0)	0.116
Others	45 (26.5)	8 (23.5)	16 (39.0)	13 (22.8)	3 (13.0)	4 (40.0)	1 (20.0)	0.221

* mother’s systemic diseases: asthma, congenital heart defect, cardiac rhythm disturbances, thyroid diseases, cancer, rheumatologic diseases, mental health problems, and intellectual disability. Significant differences are in bold.

**Table 3:** Perinatal, neonatal, and infant-related risk factors in various types of CP.

	Total Group	Hemiplegia	Diplegia	Tetraplegia	Mixed Form	Extrapyramidal Form	Ataxic Form	*p*
	*n* = 181	*n* = 39	*n* = 44	*n* = 57	*n* = 25	*n* = 10	*n* = 6	
Mode of delivery, *n* (%)								0.648
(missing data *n* = 5)								
Normal vaginal delivery	93 (52.8)	23 (60.5)	21 (48.8)	27 (48.2)	15 (62.5)	4 (40.0)	3 (60.0)	
Caesarean section	83 (47.2)	15 (39.5)	22 (51.2)	29 (52.7)	9 (39.1)	6 (60.0)	2 (40.0)	
Birth weight (kg), M ± SD	2.4 ± 1.0	3.0 ± 0.7	1.9 ± 0.9	2.3 ± 0.9	2.7 ± 1.0	2.4 ± 1.2	2.8 ± 1.2	**<0.001**
Birth weight, *n* (%)								**<0.001**
(missing data *n* = 4)								
<1000 g, ELBW	16 (9.0)	0 (0.0)	5 (11.4)	5 (8.8)	3 (12.5)	2 (20.0)	1 (16.7)	
1000–1499 g, VLBW	25 (14.1)	1 (2. 8)	15 (34.1)	9 (15.8)	0 (0.0)	0 (0.0)	0 (0.0)	
1500–2499 g, LBW	45 (25.4)	6 (16.7)	14 (31.8)	17 (29.8)	4 (16.7)	3 (30.0)	1 (16.7)	
2500–4000 g,	85 (48.0)	28 (77. 8)	9 (20.4)	26 (45.6)	15 (62.5)	4 (40.0)	3 (50.0)	
>4000 g	6 (3.4)	1 (2. 8)	1 (2.3)	0 (0.0)	2 (8.3)	1 (10.0)	1 (16.7)	
Apgar score at 1st minute,								**0.001**
*n* (%) (missing data *n* = 5)								
0–3	42 (23.9)	4 (11.1)	8 (18.2)	18 (32.1)	6 (25.0)	5 (50.0)	1 (16.7)	
4–7	58 (32.9)	5 (13.9)	23 (52.3)	19 (33.9)	6 (25.0)	3 (30.0)	2 (33.3)	
8–10	76 (43.2)	27 (75. 0)	13 (29.5)	19 (33.9)	12 (50.0)	2 (20.0)	3 (50.0)	
Apgar score at 5th minute,								**0.047**
*n* (%) (missing data *n* = 50)								
0–3	17 (13.0)	2 (6.9)	1 (3.3)	5 (12.5)	6 (33.3)	2 (25.0)	1 (16.7)	
4–7	48 (36.6)	6 (20.7)	16 (53.3)	18 (45.0)	4 (22.2)	4 (50.0)	0 (0.0)	
8–10	66 (50.4)	21 (72.4)	13 (43.3)	17 (42.5)	8 (44.4)	2 (25.0)	5 (83.3)	
Respiratory failure,	112 (69.1)	11 (34.4)	34 (82.9)	44 (81.5)	13 (65.0)	7 (70.0)	3 (60.0)	**<0.001**
*n* (%) (missing data *n* = 19)								
Infections, *n* (%)	103 (63.2)	14 (42.4)	27 (65.8)	40 (74.1)	15 (75.0)	4 (40.0)	3 (60.0)	**0.029**
(missing data *n* = 18)								
Intraventricular bleeding,	95 (58.6)	7 (21.9)	32 (78.0)	39 (72.2)	10 (50.0)	6 (60.0)	1 (20.0)	**<0.001**
*n* (%) (missing data *n* = 19)								
Neonatal convulsions,	46 (26.9)	0 (0.0)	8 (19.0)	25 (47.2)	8 (34.8)	3 (30.0)	2 (33.3)	**<0.001**
*n* (%) (missing data *n* = 10)								

ELBW—extremely low birth weight; VLBW—very low birth weight; LBW—low birth weight; Significant differences are in bold.

## Data Availability

The data presented in this study are available on request in the Department of Pediatric Neurology, School of Medicine in Katowice, Medical University of Silesia in Katowice, Poland. The data are not publicly available due to privacy restrictions.
